# Use of dental prostheses and negative self-perception of oral health in climacteric women: prevalence and associated factors

**DOI:** 10.61622/rbgo/2026rbgo64

**Published:** 2026-09-23

**Authors:** Vivian Cristina Silva Santos, Alenice Aliane Fonseca, Mônica Thaís Soares Macedo, Ronilson Ferreira Freitas, Luiza Augusta Rosa Rossi-Barbosa, Luciana Colares Maia, Antônio Prates Caldeira, Josiane Santos Brant Rocha

**Affiliations:** 1 Universidade Estadual de Montes Claros Montes Claros MG Brazil Universidade Estadual de Montes Claros, Montes Claros, MG, Brazil.; 2 Universidade Federal de Minas Gerais Belo Horizonte MG Brazil Universidade Federal de Minas Gerais, Belo Horizonte, MG, Brazil.; 3 Universidade Federal do Amazonas Manaus AM Brazil Universidade Federal do Amazonas, Manaus, AM, Brazil.

**Keywords:** Oral health, Climacteric, Self-perception, Dental prosthesis, Public health, Surveys and questionnaires

## Abstract

**Objective:**

To analyze the prevalence and factors associated with the use of dental prostheses and negative Self-Perception of Oral Health (SPOH) with sociodemographic, behavioral, and clinical factors in climacteric women.

**Methods:**

An epidemiological, cross-sectional, and analytical study was conducted with 757 climacteric women. The use of dental prostheses was assessed by self-report, and negative SPOH by the Oral Health Impact Profile (OHIP-14). Independent variables included sociodemographic, behavioral, and clinical factors. Associations were investigated using crude and adjusted Poisson regression.

**Results:**

Seven hundred fifty-seven climacteric women were evaluated, of which 43.9% reported negative SPOH and 48.7% used dental prostheses. The use of prostheses was associated with postmenopause (PR: 2.77; 95% CI: 2.12-3.68; p<0.001), marital status (PR: 1.16; 95% CI: 1.15-1.42; p=0.019), polypharmacy (PR: 1.24; 95% CI: 1.18-1.54; p=0.003), tobacco use (PR: 1.34; 95% CI: 1.09-1.65; p<0.001), hypertension (PR: 1.37; 95% CI: 1.11-1.69; p<0.001), osteoarticular diseases (PR: 1.29; 95% CI: 1.03-1.61; p<0.001), osteoporosis (PR: 1.33; 95% CI: 1.05-1.68; p<0.001), and sarcopenia (PR: 1.21; 95% CI: 1.18-1.50; p=0.004). Negative SPOH was associated with polypharmacy (PR: 1.33; 95% CI: 1.00-1.74; p=0.008), tobacco use (PR: 1.28; 95% CI: 0.99-1.66; p=0.017), osteoarticular diseases (PR: 1.25; 95% CI: 1.16-1.63; p=0.042), depression (PR: 1.69; 95% CI: 1.13-2.52; p=0.001), anxiety (PR: 1.99; 95% CI: 1.30-3.04; p<0.001), and intense climacteric symptoms (PR: 3.73; 95% CI: 2.36-6.30; p<0.001).

**Conclusion:**

A high prevalence of dental prosthesis use and negative SPOH was found among climacteric women, associated with sociodemographic, behavioral, and clinical factors. Health strategies should prioritize targeted interventions based on these determinants.

## Introduction

Achieving healthy aging requires a comprehensive understanding of the population's health status and the development of integrated care strategies that encompass health promotion, disease prevention, treatment, and rehabilitation.^(1,2)^ In the context of female aging, the climacteric phase is recognized as a physiological stage that begins with the initial signs of ovarian failure and marks the transition from the reproductive to the non-reproductive phase of a woman's life.^(3)^ Typically beginning around the age of 40 and extending up to approximately 65 years, this phase is characterized by significant hormonal fluctuations, particularly a decline in estrogen levels, which can affect various aspects of health.^(4)^ The climacteric phase encompasses three stages: perimenopause, menopause, and postmenopause. Menopause is defined as the absence of menstruation for 12 consecutive months, typically occurring between the ages of 48 and 51, and is classified as early if it occurs before age 40 and late if it occurs after age 55.^(4)^

During this transition, many women experience a range of significant changes, including physical, vasomotor, emotional, and cognitive alterations. Notable among these are hot flashes, insomnia, mood disturbances, osteoporosis, and an increased risk of cardiovascular diseases, making this period particularly challenging and potentially exerting a profound impact on quality of life.^(3)^ These changes also extend to oral health, primarily due to the decline in estrogen levels. Different oral health issues may arise, including alveolar bone resorption, atrophic gingivitis, jaw osteoporosis, xerostomia, hyposalivation, burning mouth syndrome, and periodontal diseases, all of which are frequently associated with tooth loss.^(5)^

Tooth loss, in turn, can have a detrimental effect on the quality of life of climacteric women, leading to functional, aesthetic, and psychosocial impairments. The use of dental prostheses has become a widely adopted strategy to mitigate these effects, playing a crucial role in the aesthetic, functional, and phonetic rehabilitation of these women, as well as supporting the maintenance of adequate protein intake, thereby reducing the risk of sarcopenia and frailty.^(6)^ However, despite their benefits, dental prostheses—particularly removable ones—may have drawbacks and are often associated with negative Self-Perception of Oral Health (SPOH).^(7)^

Epidemiological studies underscore the high prevalence of dental prosthesis use among climacteric women, particularly in older age groups. In a study conducted among postmenopausal women, 43.9% had fewer than 20 remaining teeth, and 13.9% were completely edentulous, highlighting the increased demand for oral rehabilitation.^(8)^ Moreover, chronic conditions such as diabetes and osteoporosis—especially in cases of low bone mineral density—have been associated with a higher prevalence of prosthesis use.^(8)^ Additionally, a study conducted in India reported that women were 2.22 times more likely to exhibit negative SPOH, particularly in socioeconomically disadvantaged populations.^(9)^

Although studies have examined the use of dental prostheses and SPOH, most have not specifically focused on climacteric women.^(10)^ Given the physiological and psychosocial changes inherent to this inevitable stage in a woman's life,^(3)^ research addressing oral health in this population is both timely and relevant, as it can assist in the development of educational strategies—particularly within the context of primary care—aimed at meeting the specific needs of this group.

In light of the above, the present study aimed to analyze the prevalence of dental prosthesis use and negative self-perception of oral health, as well as their associations with sociodemographic, behavioral, and clinical factors among women in the climacteric period.

## Methods

This is a cross-sectional, analytical and epidemiological study involving climacteric women receiving care in Primary Health Care. The study adhered to the criteria outlined in the *Strengthening the Reporting of Observational Studies in Epidemiology* (STROBE) checklist for the reporting of observational research.^(11)^ All participants signed two copies of the Informed Consent Form (ICF), one being kept by the research team and the other given to the woman.

The investigation was carried out within the Family Health Strategies (ESFs) program, which serve as the main primary health care units, with a focus on disease prevention, health promotion, improving quality of life, and reducing health risks. Each ESF is staffed by a multidisciplinary team along with community health agents, who conduct monthly home visits to map and monitor the population served. As of 2024, the municipality of Montes Claros, Minas Gerais, had 166 ESF units distributed across urban and rural areas, with a target population of 54,964 climacteric women registered in these units.

To estimate the minimum sample size, the formula for cross-sectional studies with a finite population was applied. An estimated prevalence of 50% was used for the outcome of interest, given that this investigation is part of a larger project evaluating multiple outcomes and because this value yields the largest required sample size. A 95% confidence level and a 5% margin of error were assumed, resulting in a minimum required sample of 681 women. To account for potential losses, an additional 10% was added, bringing the final sample size to 757 participants.

Women aged ≥40 years who were receiving care from the ESFs and were in adequate physical condition to undergo physical assessments were included in the study. Exclusion criteria comprised women diagnosed with neurological or degenerative conditions, such as stroke or dementia, as well as pregnant women, postpartum women, bedridden individuals, and those with severe disabling diseases.

Data were collected at the selected ESF units between April and August 2024 by a multidisciplinary team comprising professionals and students from the fields of dentistry, nursing, medicine, and physical education. All team members underwent training and calibration to ensure the standardization and quality of the collected data. Prior to the official data collection, a pilot study was conducted at one ESF unit with women from the target age group to test the questionnaire and evaluate interviewer performance. No adjustments to the data collection instrument were required. Following this phase, the main fieldwork was initiated. All participants provided written informed consent.

The outcome variables of the study were dental prosthesis use and Self-Perception of Oral Health (SPOH). Dental prosthesis use was assessed using the question: "Do you use a dental prosthesis?" with response options of "Yes" or "No," following the approach described by Venegas-Sanabria et al.^(12)^ SPOH was measured using the Oral Health Impact Profile (OHIP), originally developed by Slade and Spencer^(13)^ in Australia, with the full version comprising 49 items (OHIP-49). Subsequently, an abbreviated version with 14 items (OHIP-14).^(14)^ This instrument assesses the impact of oral health conditions on the functional and psychosocial aspects of life.^(15)^ Responses are recorded on a five-point Likert scale: never (0), rarely (1), sometimes (2), frequently (3), and always (4), yielding a total score ranging from 0 to 56 points, with higher scores indicate greater negative impact of oral health on quality of life and poorer self-perception of oral health.^(15)^ For analytical purposes, the OHIP-14 scores were dichotomized based on the sample median, a strategy commonly used in epidemiological studies to facilitate interpretation and comparison between groups with lower and higher perceived impact of oral health on quality of life.^(16)^

The independent variables included sociodemographic, behavioral, and clinical factors. Sociodemographic characteristics encompassed: climacteric phase, categorized according to participants’ age as premenopause (40 to 51 years) and postmenopause (over 51 years);^(4)^ marital status, dichotomized as having a partner (married or in a stable union) or not having a partner (single, divorced/separated, or widowed); self-reported race/skin color (white, Black, mixed-race, Indigenous, or Asian), which was subsequently grouped as white (those identifying as white), mixed-race (those identifying as mixed-race, Indigenous, or Asian), and Black (those identifying as Black). Household income was expressed in minimum wages and categorized based on the sample median (BRL 1,000.00), dichotomized as less than or equal to BRL 1,000.00 and greater than BRL 1,000.00.

The behavioral variables included polypharmacy (yes or no), defined as the use of five or more medications.^(17)^ Tobacco use was classified as being a smoker (currently smokes), former smoker (used to smoke but does not currently smoke), and non-smoker (has never smoked). Alcohol consumption was categorized as current consumer (currently drinks), former consumer (previously consumed alcohol regularly but has since stopped), and non-consumer (has never consumed alcohol).

The clinical variables included self-reported diagnoses of diabetes mellitus, systemic arterial hypertension (SAH), osteoarticular diseases, and osteoporosis, obtained through the question: "Has a doctor ever told you that you have…?" with dichotomous response options (yes/no).

The risk of sarcopenia was assessed using the SARC-CalF, which integrates the Strength, Assistance with walking, Rise from a chair, Climb stairs, and Falls (SARC-F) questionnaire with calf circumference (CC) measurement. The SARC-F, developed by Malmstrom and Morley,^(18)^ is a rapid screening tool that evaluates muscle strength, mobility, ability to rise from a chair, stair climbing, and fall history, yielding a total score ranging from 0 to 10. The SARC-CalF, adapted for use in Brazil,^(19)^ incorporates CC to enhance sensitivity in sarcopenia detection. CC is measured at the maximum circumference of the dominant calf using a measuring tape, with values ≤33 cm indicating a higher risk. The total SARC-CalF score ranges from 0 to 20 points. In the SARC-F, each item is scored from 0 to 2, where 0 indicates no difficulty, 1 indicates moderate difficulty, and 2 indicates severe difficulty or inability. For the falls item, 0 points indicate no falls in the past year, 1 point indicates 1–3 falls, and 2 points indicate 4 or more falls. When CC is ≤33 cm, an additional 10 points are added, reflecting an increased likelihood of sarcopenia. A final score ≥11 suggests a high risk for the condition.^(19)^

Mental health was assessed using the Depression, Anxiety, and Stress Scale – 21 items (DASS-21), the abbreviated version of the DASS-42 instrument,^(20)^ The scale comprises 21 items divided into three subscales-depression, anxiety, and stress—with responses scored on a Likert scale from 0 (did not apply) to 3 (applied very much). The score for each subscale is summed and then multiplied by two to ensure equivalence with the DASS-42, yielding scores ranging from 0 to 42. Severity classifications are as follows: Depression—normal (0–9), mild (10–13), moderate (14–20), severe (21–27), extremely severe (28–42); Anxiety—normal (0–7), mild (8–9), moderate (10–14), severe (15–19), extremely severe (20–42); Stress—normal (0–14), mild (15–18), moderate (19–25), severe (26–33), extremely severe (34–42). For the purposes of analysis, severity levels were grouped into three categories: normal-mild, moderate-severe, and extremely severe, with the latter category maintained as a distinct group.

Climacteric symptoms were assessed using the Menopause Rating Scale (MRS), developed to measure the severity of menopausal symptoms and their impact on quality of life.^(21)^ The scale comprises 11 items divided into three dimensions: somatic symptoms (hot flashes, cardiac discomfort, joint/muscle pain, sleep disturbances), psychological symptoms (depressed mood, irritability, anxiety, physical/mental exhaustion), and urogenital symptoms (sexual problems, urinary symptoms, vaginal dryness). Each item is rated on a Likert scale ranging from 0 (asymptomatic), 1 (mild), 2 (moderate), 3 (severe), to 4 (very severe), with a total score ranging from 0 to 44, calculated by summing the scores across all dimensions.

Initially, descriptive analyses of all investigated variables were performed using simple and relative frequencies. Subsequently, bivariate analyses were conducted between the dependent variables (dental prosthesis use and SPOH) and each independent variable using Poisson regression with robust variance. Crude and adjusted Prevalence Ratios (PR) were estimated, along with their corresponding 95% confidence intervals. Variables with a p-value ≤0.20 were selected for inclusion in the multiple analysis. Multiple analysis was then conducted using stepwise forward Poisson regression to assess the PR for prosthesis use and SPOH. Only variables demonstrating a statistically significant association with the outcome at the 5% level (p≤0.05) were retained in the final model. All analyses were performed using the Statistical Package for the Social Sciences (SPSS), version 20.0.

This study is part of a larger project entitled "Assessment of the Health Conditions of Climacteric Women: A Longitudinal Study," approved by the Research Ethics Committee of *Faculdades Integradas Pitágoras de Montes Claros* - Minas Gerais, Brazil, under approval number 6.742.243.

## Results

The sample consisted of 757 climacteric women, the majority of whom were in the postmenopausal phase. The prevalence of dental prosthesis use in the study population was 48.7%, while 43.9% reported negative Self-Perception of Oral Health (SPOH). The sociodemographic profile of the participants indicated that most had a partner, identified as mixed-race, and reported a household income below the median. Regarding behavioral factors, the majority did not report polypharmacy, had never smoked, and a considerable proportion reported alcohol consumption. With respect to clinical factors, most women did not present with conditions such as diabetes, hypertension, osteoarticular diseases, osteoporosis, sarcopenia, depression, anxiety, stress, or severe menopausal symptoms (Table 1).

**Table 1:** Descriptive analysis of the profile of climacteric women receiving care

Variables			n(%)
Dependent variables			
	Dental prosthesis use	No	388(51.3)
		Yes	369(48.7)
	Self-perception of oral health	Positive	425(56.1)
		Negative	332(43.9)
Sociodemographic factors			
	Climacteric phase	Premenopause	254(33.6)
		Postmenopause	503(66.4)
	Marital status	Has a partner	398(52.6)
		Does not have a partner	359(47.4)
	Skin color	White	140(18.5)
		Black	156(20.6)
		Mixed-race	461(60.9)
	Income*	Above the median	290(38.3)
		Below the median	467(61.7)
Behavioral factors			
	Polypharmacy	No	591(78.1)
		Yes	166(21.9)
	Tobacco use	Non-smoker	451(59.6)
		Former smoker	262(34.6)
		Smoker	44(5.8)
	Alcohol consumption	Non-consumer	229(30.3)
		Former consumer	264(34.9)
		Consumer	264(34.9)
Clinical factors			
	Diabetes	No	614(81.1)
		Yes	143(18.9)
	Hypertension	No	379(50.1)
		Yes	378(49.9)
	Osteoarticular diseases	No	380(50.2)
		Yes	377(49.8)
	Osteoporosis	No	594(78.5)
		Yes	163(21.5)
	Sarcopenia	Not present	465(61.4)
		Suggestive of sarcopenia	292(38.6)
	Depression	Normal-Mild	482(63.7)
		Moderate-Severe	122(16.1)
		Extremely severe	153(20.2)
	Anxiety	Normal-Mild	374(49.4)
		Moderate-Severe	169(22.3)
		Extremely severe	214(28.3)
	Stress	Normal-Mild	525(69.4)
		Moderate-Severe	96(12.7)
		Extremely severe	136(18.0)
	Menopausal symptoms	Asymptomatic	132(17.4)
		Mild	125(16.5)
		Moderate	193(25.5)
		Severe	307(40.6)

* Median = BRL 1,000.00

In the bivariate analysis, the dependent variable "dental prosthesis use" was associated with the following factors: sociodemographic factors climacteric phase, marital status; behavioral factors—polypharmacy, tobacco use, alcohol consumption; and clinical factors—diabetes, hypertension, osteoarticular diseases, osteoporosis, sarcopenia, depression, anxiety, stress, and menopausal symptoms. After adjustment using the Poisson regression model, the variables that remained significantly associated with dental prosthesis use were climacteric phase (PR: 2.77; 95% CI: 2.12–3.68), marital status (PR: 1.16; 95% CI: 1.15–1.42), polypharmacy (PR: 1.24; 95% CI: 1.18–1.54), tobacco use (PR: 1.34; 95% CI: 1.09–1.65), hypertension (PR: 1.37; 95% CI: 1.11–1.69), osteoarticular diseases (PR: 1.29; 95% CI: 1.03–1.61), osteoporosis (PR: 1.33; 95% CI: 1.05–1.68), and sarcopenia (PR: 1.21; 95% CI: 1.18–1.50) (Table 2).

**Table 2:** Crude and adjusted analysis of the association between dental prosthesis use and sociodemographic, behavioral, and clinical factors among climacteric women receiving care

Variables			Crude PR (CI95%)	p-value	Adjusted PR (CI 95%)	p- value
Sociodemographic factors						
	Climacteric phase	Premenopause	1.00		1.00	
		Postmenopause	2.76 (2.12-3.66)	<0.001	2.77 (2.12-3.68)	<0.001
	Marital status	Has a partner	1.00		1.00	
		Does not have a partner	1.23 (1.01-1.50)	0.003	1.16 (1.15-1.42)	0.019
	Skin color	White	1.00			
		Black	1.05 (0.76-1.45)	0.651		
		Mixed-race	1.07 (0.83-1.41)	0.433		
	Income*	Above the median	1.00			
		Below the median	1.494 (1.273-1.752)	0.000		
Behavioral factors						
	Polypharmacy	No	1.00		1.00	
		Yes	1.27 (1.01-1.58)	0.001	1.24 (1.18-1.54)	0.003
	Tobacco use	Non-smoker	1.00		1.00	
		Former smoker	1.36 (1.11-1.67)	<0.001	1.34 (1.09-1.65)	<0.001
		Smoker	1.08 (0.67-1.64)	0.612	1.07-(0.67-1.63)	0.646
	Alcohol consumption	Non-consumer	1.00			
		Former consumer	1.06 (0.84-1.35)	0.411		
		Consumer	0.78 (0.60-1.01)	0.009		
Clinical factors						
	Diabetes	No	1.00			
		Yes	1.36 (1.07-1.70)	<0.001		
	Hypertension	No	1.00		1.00	
		Yes	1.56 (1.28-1.92)	<0.001	1.37 (1.11-1.69)	<0.001
	Osteoarticular diseases	No	1.00		1.00	
		Yes	1.56 (1.27-1.91)	<0.001	1.29 (1.03-1.61)	0.001
	Osteoporosis	No	1.00		1.00	
		Yes	1.64 (1.32-2.03)	<0.001	1.33 (1.05-1.68)	<0.001
	Sarcopenia	Not present	1.00		1.00	
		Suggestive of sarcopenia	1.30 (1.05-1.61)	<0.001	1.21 (1.18-1.50)	0.004
	Depression	Normal-Mild	1.00			
		Moderate-Severe	1.13 (0.85-1.47)	0.178		
		Extremely severe	1.19 (0.93-1.51)	0.029		
	Anxiety	Normal-Mild	1.00			
		Moderate-Severe	1.13 (0.88-1.45)	0.139		
		Extremely severe	1.12 (0.89-1.41)	0.141		
	Stress	Normal-Mild	1.00			
		Moderate-Severe	1.23 (0.92-1.62)	0.021		
		Extremely severe	1.10 (0.85-1.42)	0.243		
	Menopausal symptoms	Asymptomatic	1.00			
		Mild	1.05 (0.74-1.50)	0.678		
		Moderate	1.11 (0.81-1.53)	0.373		
		Severe	1.22 (0.92-1.65)	0.055		

PR = Prevalence Ratio; 95% CI = 95% Confidence Interval.

* Median = BRL 1,000.00

Regarding negative SPOH, the bivariate analysis indicated associations with the sociodemographic factor climacteric phase; behavioral factors—polypharmacy, tobacco use, alcohol consumption; and clinical factors—osteoarticular diseases, osteoporosis, sarcopenia, depression, anxiety, stress, and menopausal symptoms. After adjustment using the Poisson regression model, the variables that remained significantly associated were polypharmacy (PR: 1.33; 95% CI: 1.00–1.74), tobacco use (PR: 1.28; 95% CI: 0.99–1.66), osteoarticular diseases (PR: 1.25; 95% CI: 1.16–1.63), depression (PR: 1.69; 95% CI: 1.13–2.52), moderate/severe anxiety (PR: 1.80; 95% CI: 1.26–2.55), extremely severe anxiety (PR: 1.99; 95% CI: 1.30–3.04), and mild (PR: 1.88; 95% CI: 1.06–3.42), moderate (PR: 1.85; 95% CI: 1.10–3.27), and severe (PR: 3.73; 95% CI: 2.36–6.30) menopausal symptoms (Table 3).

**Table 3:** Crude and adjusted analysis of the association between negative self-perception of oral health and sociodemographic, behavioral, and clinical factors among climacteric women receiving care

Variables			Crude PR (CI95%)	p-value	Adjusted PR (CI95%)	p-value
Sociodemographic factors						
	Climacteric phase	Premenopause	1.00			
		Postmenopause	1.22 (0.94-1.61)	0.074		
	Marital status	Has a partner	1.00	0.347		
		Does not have a partner	1.10 (0.86-1.40)			
	Skin color	White	1.00			
		Black	0.91 (0.61-1.37)	0.606		
		Mixed-race	1.04 (0.76-1.46)	0.734		
	Income*	Above the median	1.00			
		Below the median	1.220 (1.025-1.452)	0.025		
Behavioral factors						
	Polypharmacy	No	1.00		1.00	
		Yes	1.35 (1.02-1.77)	0.005	1.33 (1.00-1.74)	0.008
	Tobacco use	Non-smoker	1.00		1.00	
		Former smoker	1.31 (1.01-1.69)	0.010	1.28(0.99-1.66)	0.017
		Smoker	1.44 (0.86-2.27)	0.050	1.42 (0.85-2.24)	0.051
	Alcohol consumption	Non-consumer	1.00			
		Former consumer	1.18 (0.86-1.63)	0.212		
		Consumer	1.32 (0.97-1.81)	0.029		
Clinical factors						
	Diabetes	No	1.00			
		Yes	1.01 (0.73-1.36)	0.929		
	Hypertension	No	1.00			
		Yes	0.94 (0.74-1.21)	0.609		
	Osteoarticular diseases	No	1.00		1.00	
		Yes	1.34 (1.05-1.72)	0.004	1.25 (1.16-1.63)	0.042
	Osteoporosis	No	1.00			
		Yes	1.33 (1.00-1,75)	0.008		
	Sarcopenia	Not present	1.00			
		Suggestive of sarcopenia	1.18 (0.90-1.53)	0.126		
	Depression	Normal-Mild	1.00		1.00	
		Moderate-Severe	1.73 (1.24-2.39)	<0.001	1.26 (0.87-1.83)	0.121
		Extremely severe	2.44 (1.85-3.21)	<0.001	1.69 (1.13-2.52)	0.001
	Anxiety	Normal-Mild	1.00		1.00	
		Moderate-Severe	2.00 (1.43-2.78)	<0.001	1.80 (1.26-2.55)	<0.001
		Extremely severe	2.74 (2.05-3.68)	<0.001	1.99 (1.30-3.04)	<0.001
	Stress	Normal-Mild	1.00			
		Moderate-Severe	1.74 (1.23-2.42)	<0.001		
		Extremely severe	1.94 (1.45-1.57)	<0.001		
	Menopausal symptoms	Asymptomatic	1.00		1.00	
		Mild	1.87 (1.06-3.41)	0.018	1.88 (1.06-3.42)	0.018
		Moderate	1.86 (1.10-3.28)	0.013	1.85 (1.10-3.27)	0.014
		Severe	3.77 (2.38-6.36)	<0.001	3.73 (2.36-6.30)	<0.001

* Median = BRL 1,000.00

## Discussion

The present study examined factors associated with dental prosthesis use and negative self-perception of oral health (SPOH) among climacteric women receiving primary care in a large city in northern Minas Gerais. A high proportion of dental prosthesis use and a high prevalence of negative SPOH were observed. The multivariate analyses also revealed significant associations between sociodemographic, behavioral, and clinical variables and the outcomes assessed.

Similarly, international studies have reported high prevalences of dental prosthesis use among climacteric women, particularly during the postmenopausal phase.^(22)^ Moreover, tooth loss has been associated with emotional and functional impairments, negatively affecting SPOH. Research indicates that climacteric women demonstrate significant prevalences of negative SPOH, with tooth loss identified as one of the key determinants, alongside other contributing factors.^(9)^ A study conducted in South Korea found that 43% of women reported negative SPOH, highlighting the female gender as being more vulnerable to this condition.^(23)^

Although dental prostheses are essential for aesthetic and functional rehabilitation, they are often associated with negative SPOH, reflecting the challenges these women face regarding comfort, aesthetics, and overall well-being.^(6,7)^ Moreover, the negative SPOH observed in this study reflects not only the clinical aspects related to tooth loss but also psychosocial and emotional factors. Additionally, particularly among postmenopausal women, tooth loss can adversely affect self-perception due to the emotional and functional impacts of missing teeth or wearing prostheses.^(22,23)^

Sociodemographic factors such as postmenopause and marital status (absence of a husband or partner) were associated with dental prosthesis use among the women in this study. Postmenopausal women demonstrated a greater need for prostheses, corroborating findings in the literature that highlight how hormonal changes and bone loss increase the prevalence of edentulism during this stage of life.^(22)^ The absence of a partner was also associated with prosthesis use, reflecting the potential influence of social support on adherence to dental care. One study indicated that a lack of emotional support from a spouse may negatively affect self-care practices, including oral health.^(24)^ These findings suggest that sociodemographic factors, such as the postmenopausal phase and marital status, play a significant role in the oral health of climacteric women. The absence of social support may contribute to poorer SPOH, while the postmenopausal phase is directly related to an increased need for dental prostheses, underscoring the importance of specialized dental care during this period.

After multivariate analysis, behavioral factors such as polypharmacy and tobacco use remained associated with both the prevalence of dental prosthesis use and negative SPOH. Women using multiple medications exhibited a higher likelihood of prosthesis use, which is consistent with findings from a previous study that identified polypharmacy as a risk factor for untreated edentulism and the need for prosthetic rehabilitation.^(25)^ The use of multiple medications—particularly analgesics, antidepressants, and antihypertensives—has been widely associated with xerostomia and reduced salivary function, both of which contribute to the deterioration of oral health and, consequently, to negative SPOH.^(26)^

Conversely, tobacco use also emerged as a risk factor associated with prosthesis use, negatively affecting SPOH and women's overall quality of life. Smokers with periodontal disease report greater difficulties in prosthesis use and poorer SPOH due to the detrimental effects of tobacco on periodontal tissues and bone resorption.^(27)^ A study involving 610 postmenopausal women in Taiwan identified a significant association between smoking and an increased number of missing teeth.^(8)^ Similarly, research conducted in Brazil and Saudi Arabia has demonstrated that smoking is associated with poorer self-perception of oral health, particularly among women, older adults, and individuals with lower educational and income levels.^(28,29)^ These findings underscore the importance of considering both behavioral and pharmacological factors when planning interventions to improve oral health in climacteric women, as polypharmacy and smoking can complicate dental treatment and rehabilitation, increase the need for dental prostheses, and exacerbate negative SPOH.

Other associated factors included clinical conditions such as hypertension, which was found to be related to dental prosthesis use among the women in this study. The literature indicates that hypertension compromises microcirculation, promoting oral inflammation and bone loss, which, in turn, can lead to tooth loss.^(30)^ In southeastern Brazil, a study also reported a high prevalence of prosthesis use among individuals with hypertension, with 58.72% using dentures and 61.01% requiring prosthetic rehabilitation.^(31)^ These findings demonstrate that hypertension is frequently associated with an elevated risk of oral diseases and tooth loss, which subsequently increases the demand for dental interventions. Therefore, the association between hypertension and prosthesis use underscores the importance of comprehensive multiprofessional follow-up to effectively manage these comorbidities.

Osteoarticular diseases were associated with both outcome variables—dental prosthesis use and negative SPOH. A study conducted among older adults in India, predominantly women, indicated that conditions such as arthritis and other osteoarticular diseases can impair oral hygiene due to limited mobility and chronic pain, which hinder proper dental care, potentially leading to tooth loss and negatively affecting self-perception of oral health.^(32)^ The significant association between these conditions suggests that the impact of joint diseases extends beyond the musculoskeletal system to also affect oral health in affected women. The limitations in oral care caused by pain and restricted movement may increase the need for dental prostheses and negatively influence these women's perception of their oral health.

Osteoporosis and sarcopenia were also consistently associated with dental prosthesis use, likely due to reductions in bone density and muscle mass, which compromise both dental structures and gingival tissues, negatively impacting oral health^25^. Women with osteoporosis, particularly in the postmenopausal phase, have an elevated risk of tooth loss due to decreased bone mineral density, which exacerbates the likelihood of edentulism.^(33)^ Regarding sarcopenia, a study conducted in Poland with women aged 68 to 92 years found that those using complete and partial dentures exhibited greater reductions in handgrip strength—a hallmark of sarcopenia—compared to women with natural dentition.^(34)^ Moreover, individuals with compromised dentition, including prosthesis users, have been shown to have a higher risk of frailty and physical disability.^(33)^ These findings underscore that osteoporosis and sarcopenia not only impair physical mobility but also affect oral health by hindering adequate oral care and contributing to the progression of oral diseases, thereby increasing the need for dental prostheses to restore oral function.

Negative SPOH among the women studied showed a strong association with depression and anxiety. These findings are consistent with previous research highlighting the adverse impact of mental disorders on oral health, contributing to a higher prevalence of dental caries and tooth loss, largely due to reduced oral hygiene and lower adherence to dental care during this stage of life.^(35)^ These results suggest that both psychological distress and menopausal changes directly affect oral health. This underscores the need for integrated approaches that address hormonal, physical, and psychosocial factors in managing oral health among climacteric women, with the aim of improving dental care and, consequently, the quality of life of this population.

This study presents some limitations that should be acknowledged. Due to its cross-sectional design, causal relationships cannot be established, and the possibility of reverse causality should be considered. The data for the variables were collected through self-report, which may introduce biases related to subjectivity or participants’ tendency to provide socially desirable responses. Nevertheless, the importance of this study should be emphasized, as its results offer valuable insights into the oral health of a frequently overlooked segment of the Brazilian population: climacteric women. The analysis of a representative sample of these women in a large urban center enabled the identification of factors associated with dental prosthesis use and negative self-perception of oral health. These findings contribute to the advancement of new research focused on health promotion strategies aimed at improving the quality of life of women, particularly during the climacteric period.

## Conclusion

The present study revealed a high prevalence of dental prosthesis use and negative self-perception of oral health among climacteric women. Factors such as being postmenopausal, absence of a partner, polypharmacy, smoking, hypertension, osteoarticular diseases, osteoporosis, and sarcopenia were found to be associated with dental prosthesis use. Negative self-perception of oral health was associated with polypharmacy tobacco use, osteoarticular diseases, depression, anxiety, and severe climacteric symptoms.

These findings reinforce the need for comprehensive and multidisciplinary care in Primary Health Care, especially for postmenopausal women, considering the impact of hormonal, behavioral, and psychosocial factors on oral health. Strengthening preventive actions, early identification of risk factors, and expanding access to dental care may contribute to improving oral health and quality of life among climacteric women.

## Data Availability

The research data are described in the article presented.
